# Shaping the Flow: Impact of Blade Shape on Performance and Hemocompatibility in Rotodynamic Blood Pumps

**DOI:** 10.1111/aor.70178

**Published:** 2026-06-07

**Authors:** Benjamin Torner, Simon Klocker, Niccolò Crescenti, Aldo J. Suria, Harald Reithofer, Michael Hübler, Daniel Zimpfer, Marcus Granegger

**Affiliations:** ^1^ Department of Cardiac and Thoracic Aortic Surgery Medical University of Vienna Vienna Austria; ^2^ University Heart and Vascular Center Hamburg University Medical Center Hamburg‐Eppendorf Hamburg Germany

## Abstract

**Objectives:**

Rotodynamic blood pumps (RBPs) must operate reliably across a wide range of clinical conditions, including off‐design operating regimes. Although blade shape is known to influence off‐design performance in conventional turbopumps, its impact on hydraulic performance and hemocompatibility in RBPs has not been systematically investigated. This study combines in silico and in vitro analyses to assess the effects of leading‐edge geometry and blade thickness on performance (pressure head‐flow rate characteristics, efficiencies) and hemocompatibility (damage index, DI) across multiple RBPs and operating conditions.

**Methods:**

Three representative RBPs, a cavopulmonary assist device, the CentriMag RBP, and a novel ventricular assist device, were investigated. Semi‐circular, elliptical, and wedge‐shaped leading edges were implemented, whereas three blade thickness configurations were analyzed.

**Results:**

Variations in leading‐edge geometry resulted in minor differences in performance (< 3 percent in efficiency) and hemocompatibility (< 3% DI) across all devices and operating conditions. In contrast, blade thickness had a pronounced effect on performance under overload off‐design operation, with thinner blades improving efficiency by up to 22%, while exhibiting only limited changes in hemocompatibility‐related metrics (< 7% DI). The observed efficiency differences were primarily attributed to decreased friction losses caused by less channel blockage with thinner impeller blades.

**Conclusions:**

Overall, impeller blade shape plays a secondary role in hemocompatibility within the investigated pump types and operating conditions. This finding suggests that leading‐edge optimization may be deprioritized relative to other hemocompatibility‐critical geometry parameters in RBP design. Blade thickness, however, may lead to unfavorable channel blockage effects and therefore needs to be optimized with respect to manufacturing constraints and hydraulic performance.

## Graphical abstract

Blade shapes (leading‐edge geometry and thickness) in rotodynamic blood pumps were systematically evaluated. Thinner blades improve pump efficiency by reducing blockage effects, while neither blade thickness nor leading‐edge geometry shows clear hemocompatibility benefits. Overall, blade shape has a limited impact on hemocompatibility, indicating it is a secondary design parameter.

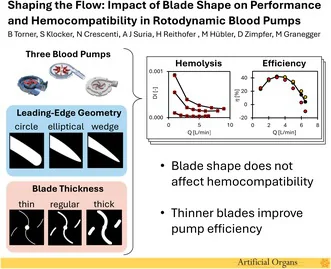

## 1 Background

Rotodynamic blood pumps (RBPs) are widely used for mechanical circulatory support [1], including ventricular assist devices (VADs) and extracorporeal membrane oxygenation systems. A key requirement of RBPs is that they operate reliably across a broad range of physiological flow conditions. This requires sufficient hydraulic performance, including pressure head and efficiency, and the preservation of high hemocompatibility. Various studies [2, 3, 4] have shown that RBPs operate across a broad range of flow conditions in clinical practice, frequently under off‐design conditions that reduce efficiencies and increase the hemolytic potential [4, 5]. Consequently, design strategies that enhance off‐design performance without compromising hemocompatibility are essential.

Several approaches have been proposed in recent years to improve RBP operation under both design and off‐design conditions. As RBPs are turbopumps that generate pressure head by imparting momentum through rotating impeller blades, some studies have explored pitch‐adjustable and flexible blades [6, 7], as well as multi‐stage impeller concepts [8, 9].

Another approach can be derived from conventional turbopump design guidelines [10]. Here, variations in impeller blade shape, such as modifications of the leading‐edge geometry, are parameters influencing the performance. The underlying physics are illustrated in Figure 1, which shows the influence of different leading‐edge geometries on the performance. At off‐design conditions, deviations between inflow and blade angles may cause flow separation near the leading‐edge, reducing efficiency [10, 11]. Since reduced efficiency is associated with increased flow losses and stresses [12, 13], variations in leading‐edge geometry are hypothesized to also influence hemocompatibility. However, it remains unclear whether these mechanisms translate to RBP flows.

**FIGURE 1:**
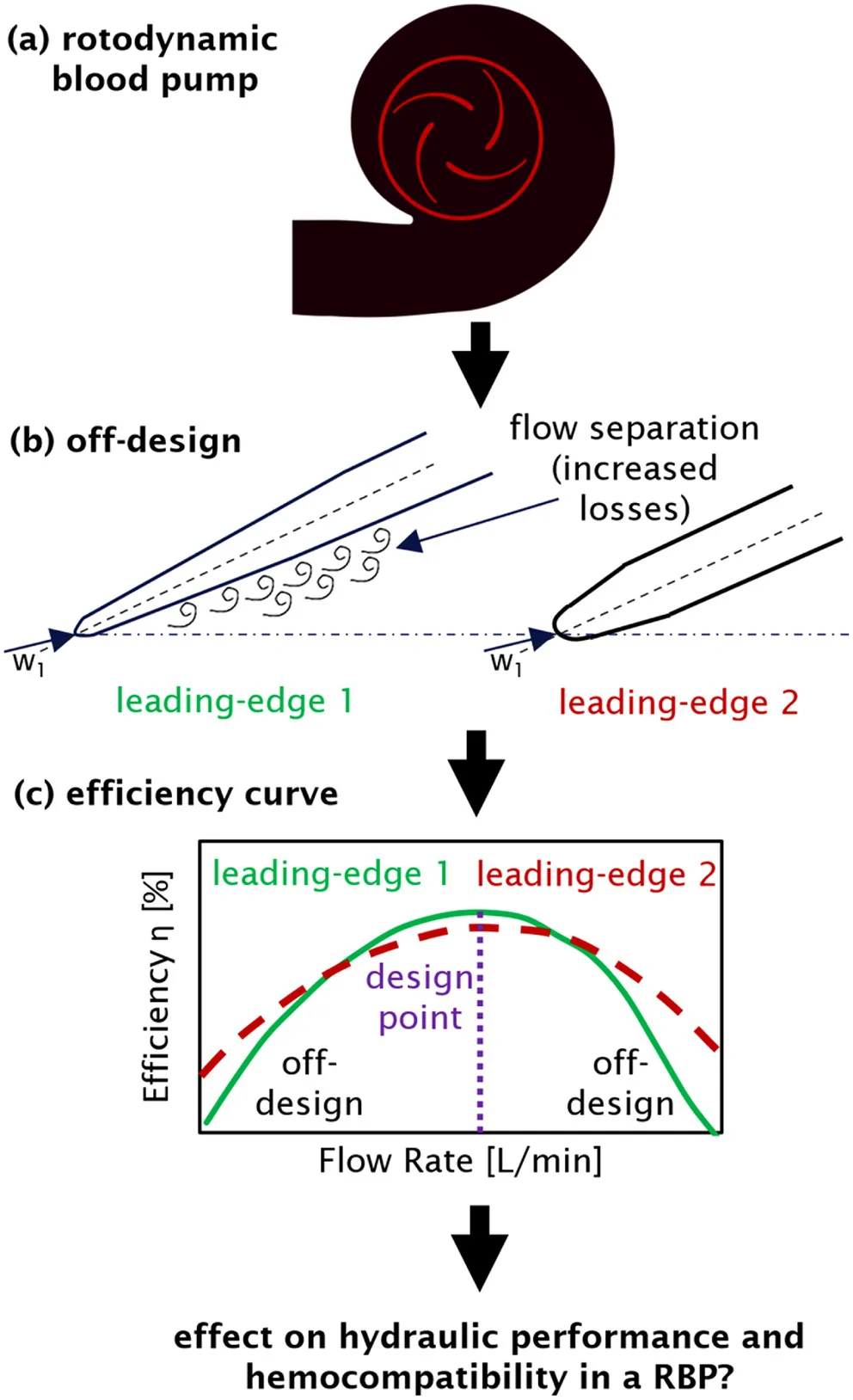
Conceptual influence of the leading‐edge geometry on the off‐design behavior of rotodynamic blood pumps (RBPs). (a) RBP with highlighted impeller blades. (b) Illustration of two leading‐edge geometries and their differing tendencies for incidence‐induced flow separation, according to the theory of Ref. [10]. (c) Expected effect of these variations on the efficiency curve.

In addition, blade thickness is another blade shape parameter that influences the off‐design behavior. The underlying physical rationale originates from airfoil theory, which shows that airfoils exhibit increased resilience to flow separation at high incidence angles as their thickness increases up to a certain thickness range [14]. Translating this effect to RBPs suggests that certain blade thicknesses may also widen the efficiency range.

However, recent analyses of the effect of blade thickness in RBPs indicate a controversial picture. In some studies [15, 16], increasing thickness led to improved hydraulic and hemolytic performance, whereas thicker blades performed worse in others [17]. Conversely, Li et al. reported that blade thickness had a marginal effect in their RBP under investigation [18]. A major limitation shared by these studies is that thickness effects were evaluated for a single pump and under a limited number of operating points, preventing generalized conclusions about thickness‐induced flow phenomena.

Taken together, it remains unclear whether specific blade shape parameters, namely leading‐edge geometry and blade thickness, govern hydraulic performance and hemocompatibility in RBPs under off‐design conditions. A comprehensive evaluation across different RBPs and operating points is required.

To address this question, this study integrates simulations with experimental validation to systematically assess how leading‐edge geometry and blade thickness affect hydraulic performance and hemocompatibility over the entire operating range. The analysis was conducted across three distinct RBPs, a cavopulmonary assist device (CPAD) developed for failing Fontan patients, the CentriMag RBP (Abbott, USA), and a centrifugal RBP developed for research purposes in VAD applications.

## 2 Methods

### 2.1 RBPs and Operating Conditions

Three RBPs were investigated in this study. These pumps differ substantially in geometry, intended application, and operating conditions. This diversity enables the assessment of blade shape configurations across a broad range of designs and operating regimes.

The first device is a CPAD developed at the Medical University of Vienna [2] (Figure 2). It is designed to deliver a pressure head of H=12mmHg at a flow rate of Q=4L/min, achieved at a rotational speed of n=2850rpm. To capture off‐design behavior, simulations were conducted over a flow range spanning from 1L/min to near zero‐head condition at 6.5L/min.

**FIGURE 2:**
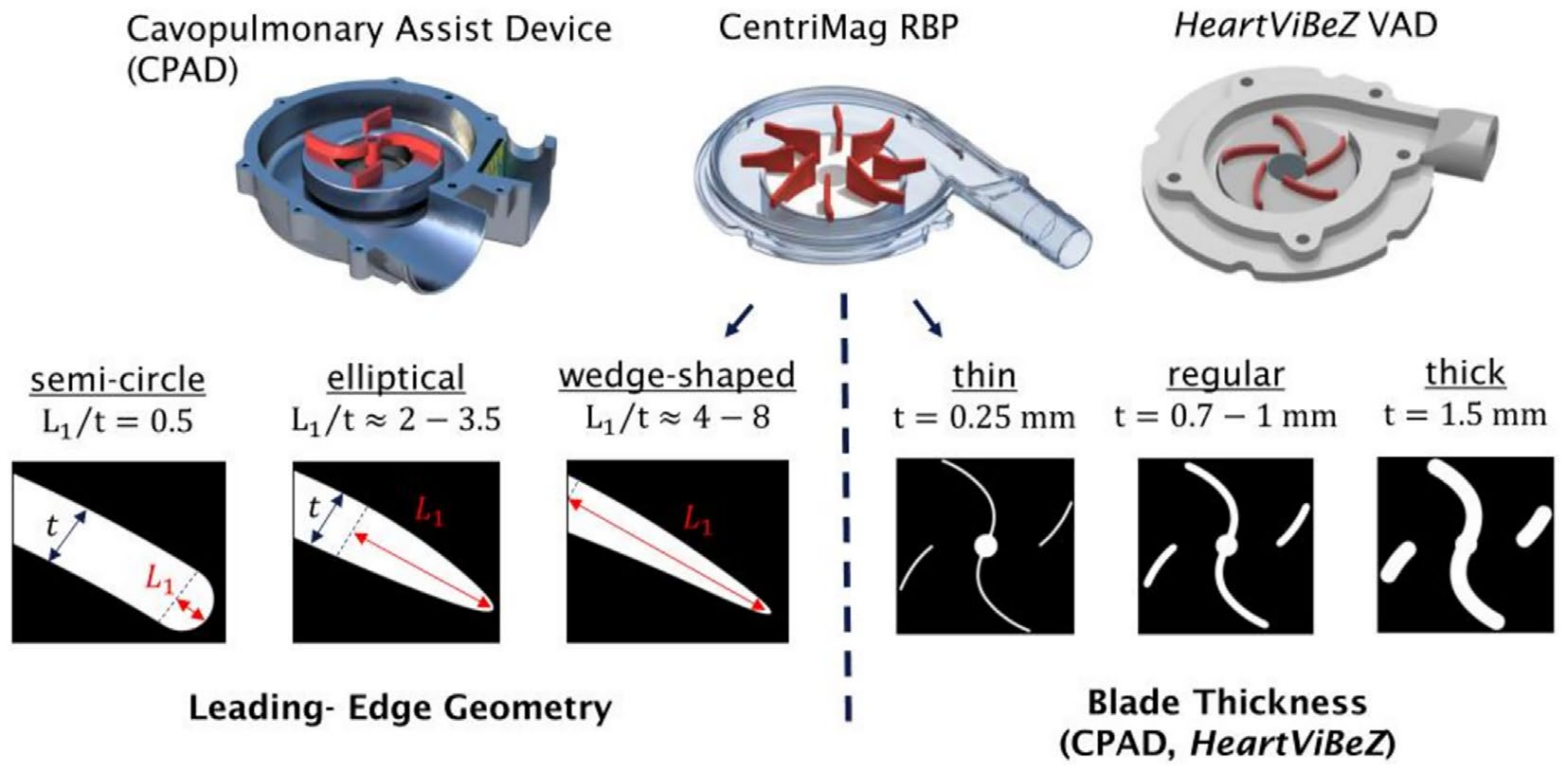
Rotodynamic blood pumps (RBPs) investigated in this study and the blade shape modifications. $L_{1}$ denotes the leading‐edge length and t is the blade thickness. VAD—ventricular assist device.

The second device is the CentriMag RBP whose geometry was reconstructed from 3D scans. The RBP was evaluated under VAD‐relevant operating conditions (H=80mmHg, Q=5L/min, n=2150rpm), comparable to values reported in Ref. [19]. Off‐design performance was assessed by varying flow rates between $\left(1-15\right)\;L/\min$, where the higher flow rate is near zero‐head condition.

The third RBP investigated is the *HeartViBeZ* VAD, a research prototype developed collaboratively by the Medical University of Vienna, the Technical University of Berlin, and ETH Zurich. This pump was included as a centrifugal VAD, designed based on classical turbomachinery principles (e.g., from Ref. [10]). The pump was designed to achieve a pressure head of 70mmHg at a flow rate of 5L/min and a rotational speed of =2650rpm. Off‐design operation was investigated over a range from 1L/min to near zero‐head condition at 8L/min.

### 2.2 Blade Shape Configurations

Three leading‐edge geometries were investigated (Figure 2). They were designed according to turbomachinery design guidelines [10, 20], which define ratios between the leading‐edge length $L_{1}$ and the blade thickness t. A semi‐circular leading‐edge with a ratio of $L_{1}/t=0.5$ was investigated as the baseline. According to the literature [10, 11], such blunt leading‐edges lead to increased separation losses under off‐design conditions. Second, an elliptical leading‐edge with $L_{1}/t\approx \left(2-3.5\right)$ was considered (CPAD: 2.7, CentriMag: 3.6, *HeartViBeZ*: 3.0), which is expected to reduce separation losses under off‐design operation [10, 20]. Finally, a wedge‐shaped leading‐edge with $L_{1}/t\approx \left(4-8\right)$ was examined (CPAD: 6.0, CentriMag: 6.0, *HeartViBeZ*: 6.0). This geometry represents a sharp design commonly associated with increased sensitivity to separation effects. Representative images are provided in Supporting Information S1.

In addition to leading‐edge geometry, three different thickness configurations were investigated for the CPAD and the *HeartViBeZ* VAD. Turbomachinery literature provides blade thickness recommendations based on structural considerations, while guidelines for hydraulic performance are lacking. Therefore, three empirical thickness configurations were investigated. The baseline blade thickness of t=0.7mm for the CPAD and t=1.0mm for the VAD was reduced to a thin configuration (t=0.25mm). In addition, a thick blade configuration corresponding to approximately twice the baseline thickness (t=1.5mm) was investigated.

Blade thickness effects were investigated using two design sets: design set A, with unchanged impeller blade channel geometry, and design set B, in which the inlet and outlet blade channel geometries were adjusted to achieve similar inlet and outlet radial velocities. Design set B was implemented by modifying the blade width at the inlet and outlet areas.

The CentriMag RBP was not included in the blade thickness study due to its open‐impeller design without a shroud, which is uncommon among RBPs. This geometry precludes a consistent implementation of design set B and would therefore limit the systematic generalizability of thickness‐related conclusions for the more commonly used shrouded RBPs.

### 2.3 Computational Fluid Dynamics Setup

The unsteady Reynolds‐averaged Navier–Stokes (URANS) equations were solved for more than 100 simulations using the commercial solver Simcenter Star‐CCM+ 2410 (Siemens Digital Industries Software, TX, USA). Turbulence was modeled using the k‐ω‐SST model [21]. Streamline curvature [22] and flow transition effects [23] were incorporated via established correction terms and transport equations.

Polyhedral meshes with prism‐layer wall resolution (10 layers, surface‐averaged *y* ^+^ < 1) were used for all simulations. The meshes contained approximately 8 million cell elements per simulation. Grid quality criteria recommended by Star‐CCM+ were satisfied [24]. Furthermore, grid uncertainty was assessed following Ref. [25] to evaluate discretization errors (see Supporting Information S2).

Second‐order spatial and temporal discretization schemes were used. A transient rotor‐stator sliding mesh approach was applied with a time step corresponding to an impeller rotation of four degrees. For the most restrictive case (CentriMag, 8 blades, 2150 rpm), the blade‐passing frequency (287 Hz) remains well below the Nyquist frequency (~1613 Hz), and approximately 11 time steps per blade‐passing period are obtained, ensuring adequate temporal resolution of unsteady blade‐tongue interactions. Simulations covered 10–30 revolutions per operating point, with time‐averaging over the final five revolutions.

Blood was modeled as a Newtonian fluid [26] (density $\rho =1050\;\mathrm{kg}/m^{3}$, dynamic viscosity μ=3.5mPas). All walls were treated with no‐slip condition. A constant flow rate with a uniform velocity profile was prescribed at the inlet boundary, while zero total pressure was imposed at the outlet boundary. Both boundaries were positioned between distances of 4–11 times the respective cannula diameter from the RBPs. Convergence was assessed by root‐mean‐square (RMS) residuals approaching 10^−4^ and stable integral quantities.

### 2.4 Metrics for Hydraulic Performance, Flow Losses, Blade Loads, and Hemocompatibility

Hydraulic performance was quantified by the pressure head H and inner efficiency η using Equations (1) and (2), based on the total pressures $p_{\mathrm{tot}}$ at the pump inlet and outlet, the flow rate Q, the impeller torque M, and the rotational speed n.

(1) $$\begin{array}{c} H=\frac{\sum_{\text{outlet}}\;p_{\mathrm{tot}}\cdot {\dot{m}}_{\text{cell}}}{\sum_{\text{outlet}}{\dot{m}}_{\text{cell}}}-\frac{\sum_{\text{inlet}}\;p_{\mathrm{tot}}\cdot {\dot{m}}_{\text{cell}}}{\sum_{\text{inlet}}{\dot{m}}_{\text{cell}}}\;\left(\text{mmHg}\right) \end{array}$$

(2) $$\eta =\frac{∆p_{\mathrm{tot}}\cdot Q}{M\cdot 2\pi n}\cdot 100\;\left[\%\right]$$

Total flow losses were determined from the difference between mechanical input and hydraulic output power:

(3) $$P_{\text{Loss}}=M\cdot 2\mathrm{\pi n}-∆p_{\mathrm{tot}}\cdot Q\;\left[W\right].$$

Blade loading was evaluated to identify stress hotspots. It was assessed by evaluating the scalar stress distribution along the blade surface from the impeller inlet to the outlet radius.

Hemolysis was assessed by the damage index DI, computed based on established power‐law formulations relating blood cell damage to scalar stress $\tau_{s}$ and exposure time t. The empirical constants $\left[C,\alpha ,\beta \right]=\left[1.8\cdot 10^{-6},1.991,0.765\right]$ were taken from Refs. [27, 28]. The damage index was evaluated using a volume‐integral formulation proposed by Ref. [29], as given in Equation (4).

(4) $$\mathrm{DI}={\left(\frac{1}{Q}\int_{\text{pump}}C^{\frac{1}{\beta }}\tau_{s}^{\frac{\alpha }{\beta }}\mathrm{dV}\;\right)}^{\beta }\left[-\right]$$

The scalar stress was defined as follows:

(5) $$\tau_{s}=\sqrt{2\mu^{2}S_{\mathrm{ij}}S_{\mathrm{ij}}+\rho \mu \varepsilon_{\mathrm{mod}}}\;\left[\mathrm{Pa}\right],$$

where $S_{\mathrm{ij}}$ denotes the rate‐of‐strain tensor, and $\varepsilon_{\mathrm{mod}}$ is a turbulence model‐related dissipation term.

### 2.5 Experimental Validation Setup

The simulation results were experimentally validated through hydraulic performance measurements for all RBPs.

CPAD measurements were conducted using a hydraulic test rig (Supporting Information S1). It featured two inflows and one outflow, representing the venae cavae to the pulmonary artery flows. The CPAD was manufactured using stereolithography (Form 3 printer, Formlabs, USA). The integrated motor with permanent magnets of the original design was replaced by an external motor, which drove the impeller via a shaft.

For the CentriMag and the *HeartViBeZ* VAD, H‐Q characteristics were obtained using a hydraulic test rig compliant with DIN ISO 9906 [30] (Figure A2, Supporting Information S1). To drive and magnetically levitate the impellers, the magnetic bearing and drive unit of the original CentriMag RBP was used. Impellers with different leading‐edge geometries were manufactured using the Form 3 printer. After post‐processing, magnets retrieved from disposable CentriMags were incorporated into the 3D‐printed impellers.

During testing, rotational speed was fixed while flow rate was increased in 0.25 L/min steps. Pressure head and flow rate were recorded for 30 s and averaged for the last 10 s. Three HQ curves were recorded per impeller and averaged at each operation point. Then the uncertainty at each point was calculated in accordance with Ref. [31].

## 3 Results

### 3.1 Influence of the Leading‐Edge Geometry

Figure 3 illustrates the influence of the leading‐edge geometry on hydraulic performance obtained from the numerical simulations. Only minor differences in pressure head and efficiency were observed between the leading‐edge configurations.

**FIGURE 3:**
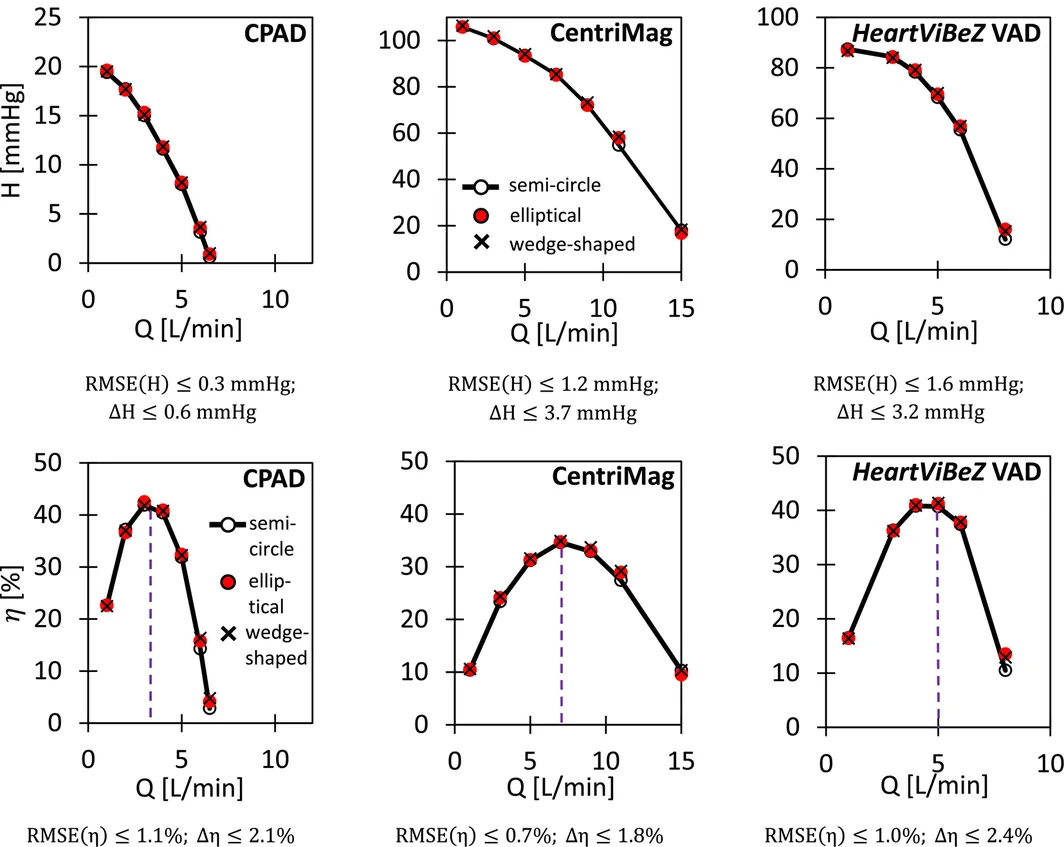
Pressure head H and efficiency η curves of the RBPs with different leading‐edges. The maximum root‐mean‐square error (RMSE) between two curves and the maximum absolute deviation are reported. The purple dotted line indicates the best efficiency point. CPAD—cavopulmonary assist device, VAD—ventricular assist device.

To quantify these differences, the root‐mean‐square error (RMSE) and the maximum absolute deviation were evaluated. For all conditions, RMSE for pressure heads remained below 1.6 mmHg and RMSE for efficiencies below 1.0%. The maximum deviations were likewise small, with less than 3.7mmHg in pressure head for the CentriMag RBP at 10L/min and less than 2.4% in efficiency for the *HeartViBeZ* VAD at 8L/min.

Experimental measurements of the hydraulic performance showed good agreement with the simulations, with maximum deviations below 10 mmHg for all pumps and operating conditions (see Supporting Information S1).

Similar trends of the leading‐edge influence were also observed in the hemolysis‐related metrics. Figure 4 displays the damage index DI for the three configurations. Across all conditions, the relative deviations remained below 3%.

**FIGURE 4:**
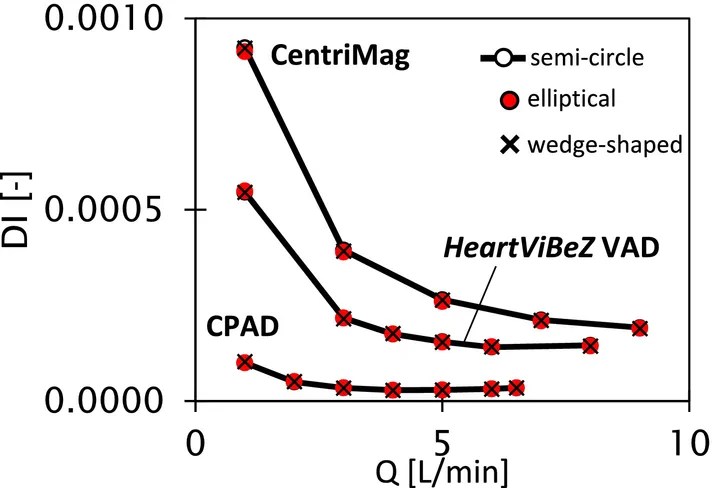
Damage index DI curves with the three leading‐edge modifications. CPAD—cavopulmonary assist device, VAD—ventricular assist device.

To characterize local flow features associated with the different leading‐edge geometries, additional analyses were performed for the *HeartViBeZ* VAD at an off‐design operating point (8L/min), where the largest efficiency differences were observed. Figure 5a visualizes regions of negative time‐averaged radial velocities in the relative frame of reference, indicating flow separation regions near the leading edge. Differences in the extent of these regions were observed between the three leading‐edge geometries.

**FIGURE 5:**
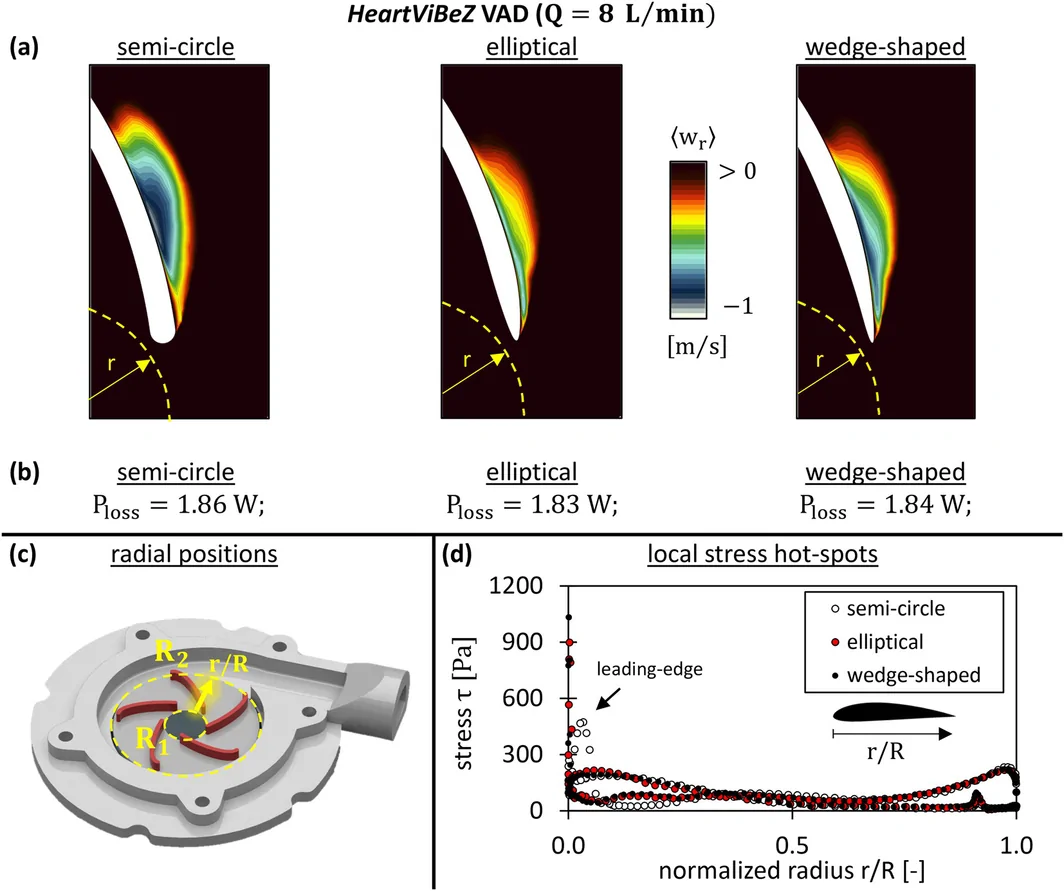
Local flow and losses in the *HeartViBeZ* VAD at Q=8L/min. (a) Visualization of flow separation behavior around the leading‐edge by the radial velocity in the relative frame of reference. (b) Flow losses integrated over the entire pump volume. (c) Schematic drawing of inlet $R_{1}\;\left(r/R=0\right)$ and outlet radius $R_{2}\;\left(r/R=1\right)$ with normalized radius r/R. (d) Progression of the local scalar stresses around the impeller blade.

Despite these local differences, the integrated flow losses within the pump remained comparable across all configurations, as shown in Figure 5b. The flow losses varied marginally between the semi‐circular, elliptical, and wedge‐shaped leading‐edge configurations.

The most pronounced differences were observed in the local stress distributions along the impeller blade. Figure 5c,d show the progression of scalar stress from the inlet radius to the outlet radius. Under off‐design conditions, the elliptical and wedge‐shaped leading edges exhibited higher peak scalar stresses in the immediate leading‐edge region compared to the semi‐circular profile. The maximum scalar stresses were approximately 470Pa for the semi‐circular, 900Pa for the elliptical, and 1030Pa for the wedge‐shaped configuration. These stress peaks were spatially confined to the leading‐edge region and exhibited similar patterns at other off‐design conditions.

### 3.2 Influence of the Blade Thickness

Figure 6 illustrates the influence of the blade thickness on the hydraulic performance of the CPAD and the *HeartViBeZ* VAD. The results shown correspond to design set A, in which blade thickness was varied while maintaining same inlet and outlet blade channel geometries. Blade thickness variations resulted in larger differences in hydraulic performance than the leading‐edge modifications. Maximum efficiency differences between thin and thick blades reached 13.4 percentage points for the VAD (8L/min) and 21.6 percentage points for the CPAD (6.5L/min).

**FIGURE 6:**
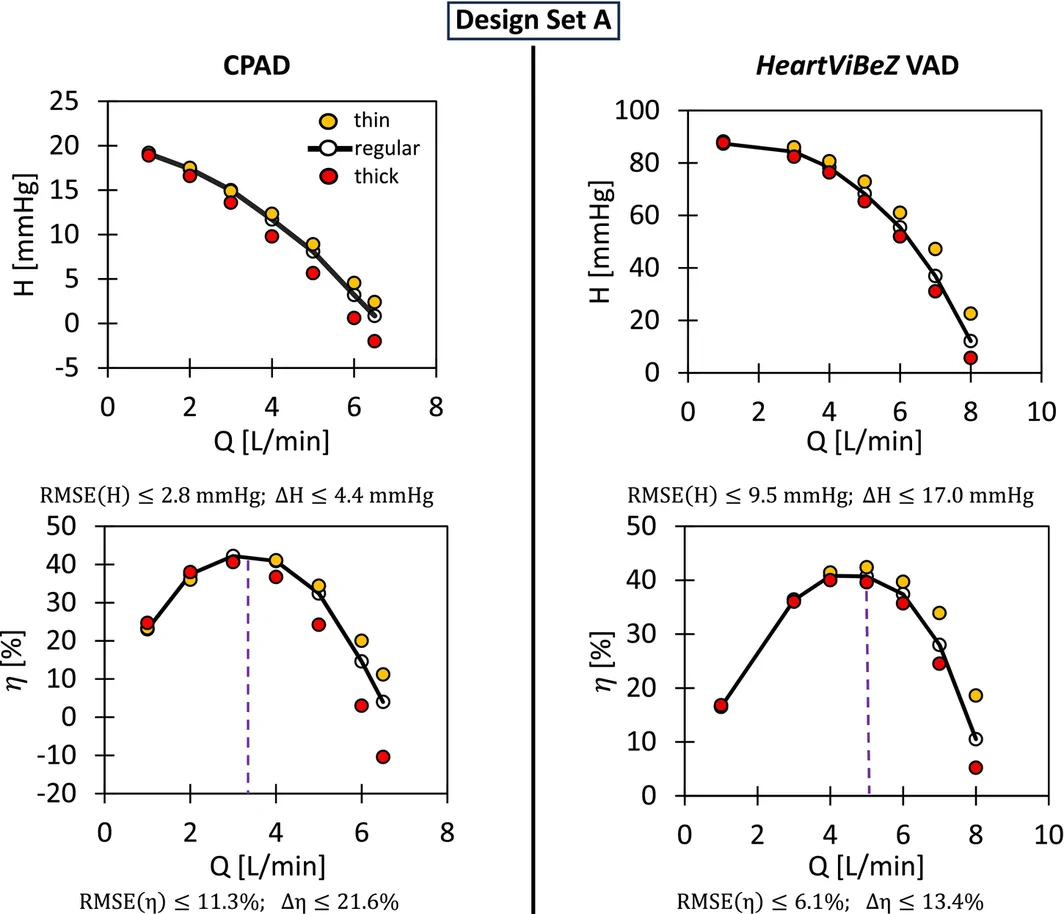
Pressure head H and efficiency η curves of the cavopulmonary assist device (CPAD) and the *HeartViBeZ* ventricular assist device (VAD) with the three thickness variations (design set A: Identical inlet and outlet blade channel geometries). The maximum root‐mean‐square error (RMSE) between two curves and the maximum absolute deviation are reported. The purple dotted line indicates the best efficiency point.

For both RBPs, deviations increased with flow rate beyond the best efficiency point. In this overload off‐design regime, thinner blades yielded higher pressure head and efficiency, whereas thicker blades exhibited a pronounced degradation in hydraulic performance.

Figure 7 shows the influence of blade thickness on hydraulic losses and hemolysis‐related metric for design set A. Differences in flow losses between the blade thickness configurations became apparent under off‐design conditions. At partial‐load operation, thinner blades exhibited similar losses for the *HeartViBeZ* VAD and slightly higher losses for the CPAD compared to thicker blades, with maximum relative deviations of up to 10.9% for the CPAD at less than 2L/min. In contrast, under overload conditions, thicker blades were associated with increased flow losses for both RBPs, with relative deviations of up to 11.6% for the CPAD at 6.5L/min.

**FIGURE 7:**
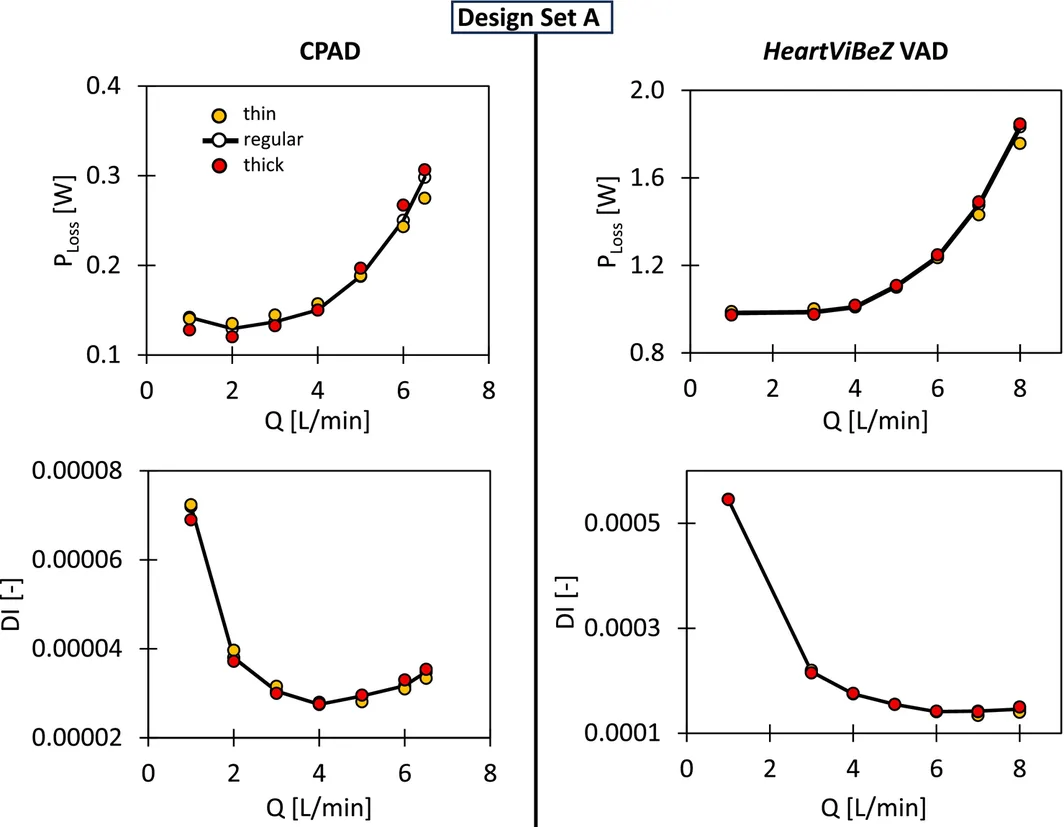
Flow losses and damage index (DI) for the cavopulmonary assist device (CPAD) and the *HeartViBeZ* ventricular assist device (VAD) with different blade thicknesses (design set A: Same inlet and outlet blade channel geometries).

Comparable relative trends were observed for the hemolysis‐related metric shown in Figure 7. At partial‐load operation, the thin blade configuration exhibited a similar or higher damage index DI at the investigated points, whereas under overload conditions it consistently showed lower values compared to the thick blade configuration. The maximum relative deviations in DI between thin and thick blades reached 6.3% for the CPAD for flow rates smaller than 3L/min, and approximately 6.5% at 6L/min for the CPAD and at 8L/min for the *HeartViBeZ* VAD.

Figure 8 presents the hydraulic performance and hemolysis‐related metric for blade thickness variations in design set B, in which the inlet and outlet blade channel areas were adjusted to achieve similar radial velocities at these positions. In contrast to design set A, differences in pressure head and efficiency between the thin, regular, and thick blade configurations were substantially reduced for both RBPs. The remaining deviations in hydraulic performance were small, with RMSE values below 0.8mmHg for the CPAD and 3.9mmHg for the HeartViBeZ VAD, and RMSE efficiency deviations remaining below 3.1%.

**FIGURE 8:**
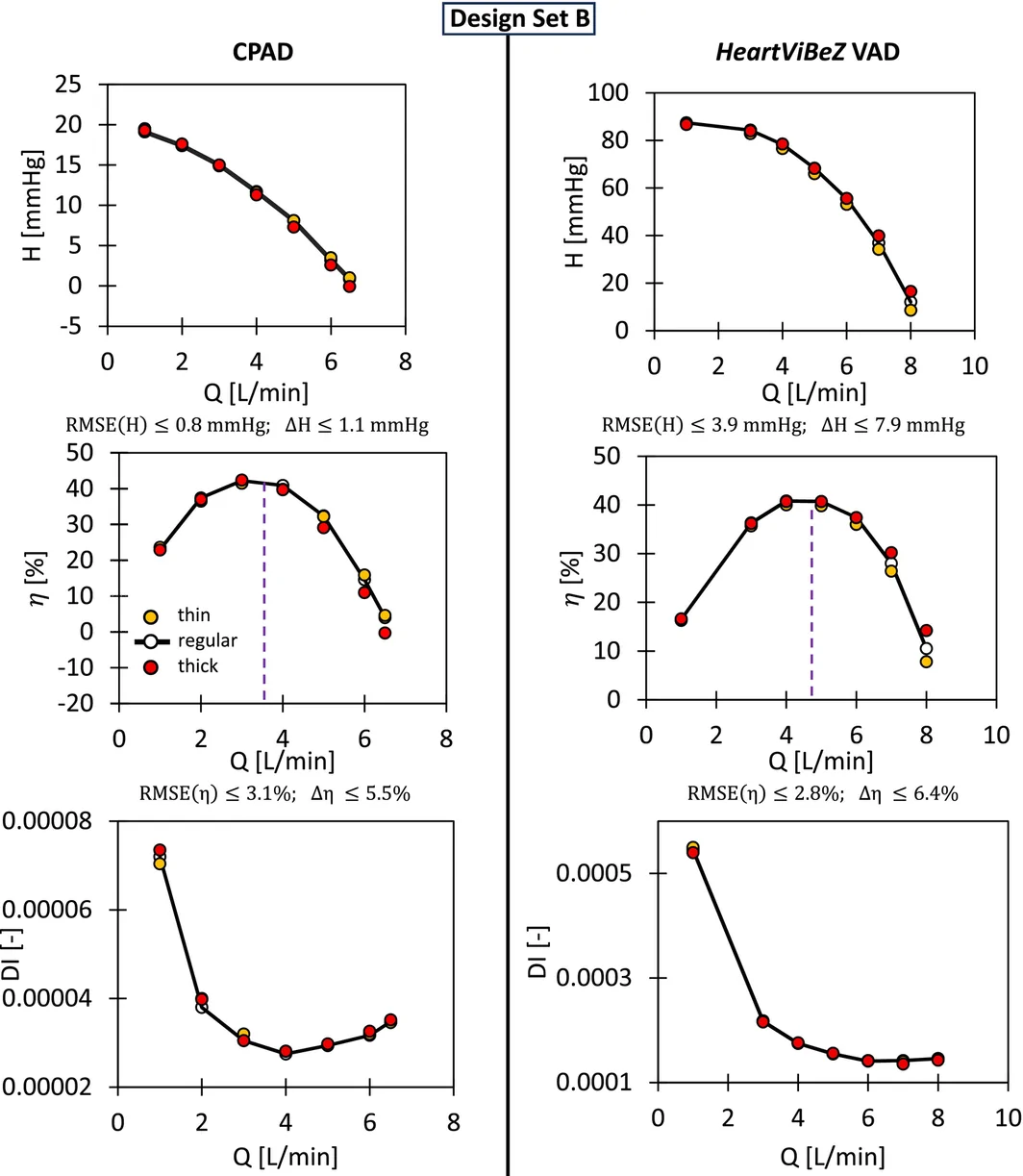
Pressure head H, efficiency η and DI curves of the cavopulmonary assist device (CPAD) and the *HeartViBeZ* VAD with different blade thicknesses (design set B: Adapted inlet and outlet channel geometries to obtain similar radial velocities). The maximum root‐mean‐square error (RMSE) between two curves and the maximum absolute deviations are reported for H and η. The purple dotted line indicates the best efficiency point.

A similar trend was observed for the hemolysis‐related metric. As shown in Figure 8, the damage index curves for the different blade thickness configurations are similar for both RBPs, with relative deviations in damage index smaller than 5% across all operating conditions.

## 4 Discussion

The objective of this study was to determine whether modifications of impeller blade shape can enhance the hydraulic performance and hemocompatibility of RBPs across wider operating ranges. To this end, systematic variations in leading‐edge geometry and blade thickness were evaluated in three RBPs.

### 4.1 Influence of Leading‐Edge Geometry

Distinct local flow patterns consistent with prior turbopump literature were observed, including differences in separation behavior associated with semi‐circular and wedge‐shaped leading edges [10]. However, these local differences did not translate into higher flow losses, nor did they alter the main flow paths responsible for pressure head generation within the impeller. Consequently, both hydraulic performance and hemolysis‐related metrics exhibited only marginal differences.

This behavior contrasts with findings reported for industrial turbopumps, where the leading‐edge profile is reported to influence flow field development and performance [10]. This apparent discrepancy may be explained by the fundamentally different flow regimes governing RBPs, compared to industrial turbopumps. The smaller characteristic dimensions of RBPs, combined with the higher blood viscosity, result in significantly lower Reynolds numbers (Re~104) [32] compared to larger‐scale industrial pumps (Re~106) [33]. In addition, industrial pumps operate at pressure heads that are one to two orders of magnitude higher than those of RBPs (e.g., in Ref. [34]), leading to stronger pressure gradients and, consequently, more pronounced flow separations within the blade channel passages. Under such conditions, leading‐edge‐induced separation may dominate loss generation, whereas in RBPs, these mechanisms are attenuated. Together, the lower Reynolds numbers and reduced pressure gradients may suppress more pronounced separation phenomena and associated losses, thereby reducing the sensitivity of hydraulic performance to leading‐edge geometry.

Despite minimal influences on global parameters, the leading‐edge geometry affected the local stresses around the impeller blades under off‐design conditions. Thinner leading‐edges (elliptical or wedge‐shaped) were associated with more pronounced, spatially confined stress hot‐spots in the immediate leading‐edge region. Importantly, these localized stress peaks occupied only a small fraction of the pump volume and contributed negligibly to the cumulative shear exposure experienced by the blood flow. This explains the absence of marked differences in the computed hemolysis metric. Nevertheless, to assess whether such localized stress hot‐spots could give rise to detectable blood damage beyond numerical predictions, hemolysis measurements would be required in future studies.

### 4.2 Influence of Blade Thickness

At off‐design operating conditions, deviations between the investigated blade thickness configurations become increasingly pronounced for design set A, with the largest differences observed in efficiency under overload conditions. These observations are consistent with blockage effects caused by thicker blades, which reduce effective flow‐through area and increase friction losses.

When the inlet and outlet radial velocities are equalized (design set B, Figure 8), the pronounced efficiency penalties observed for thicker blades are largely eliminated. For the *HeartViBeZ* VAD, this adaptation improved hydraulic performance for thicker blades. This indicates that the previously observed performance degradation is primarily attributable to blockage‐related effects rather than to intrinsic thickness‐dependent flow phenomena.

The flow visualizations in Figure 9 provide additional insight and possible explanations into the remaining differences of the thickness configurations. First, a slight reduction of the separation region is observed with increasing blade thickness from thin to thick configurations in design set B. This is consistent with trends reported in the Introduction from airfoil theory [14]. However, since similar separation‐related effects were also observed for the leading‐edge variations without detectable consequences, separation seems unlikely to represent a dominant loss mechanism for the investigated thickness configurations.

**FIGURE 9:**
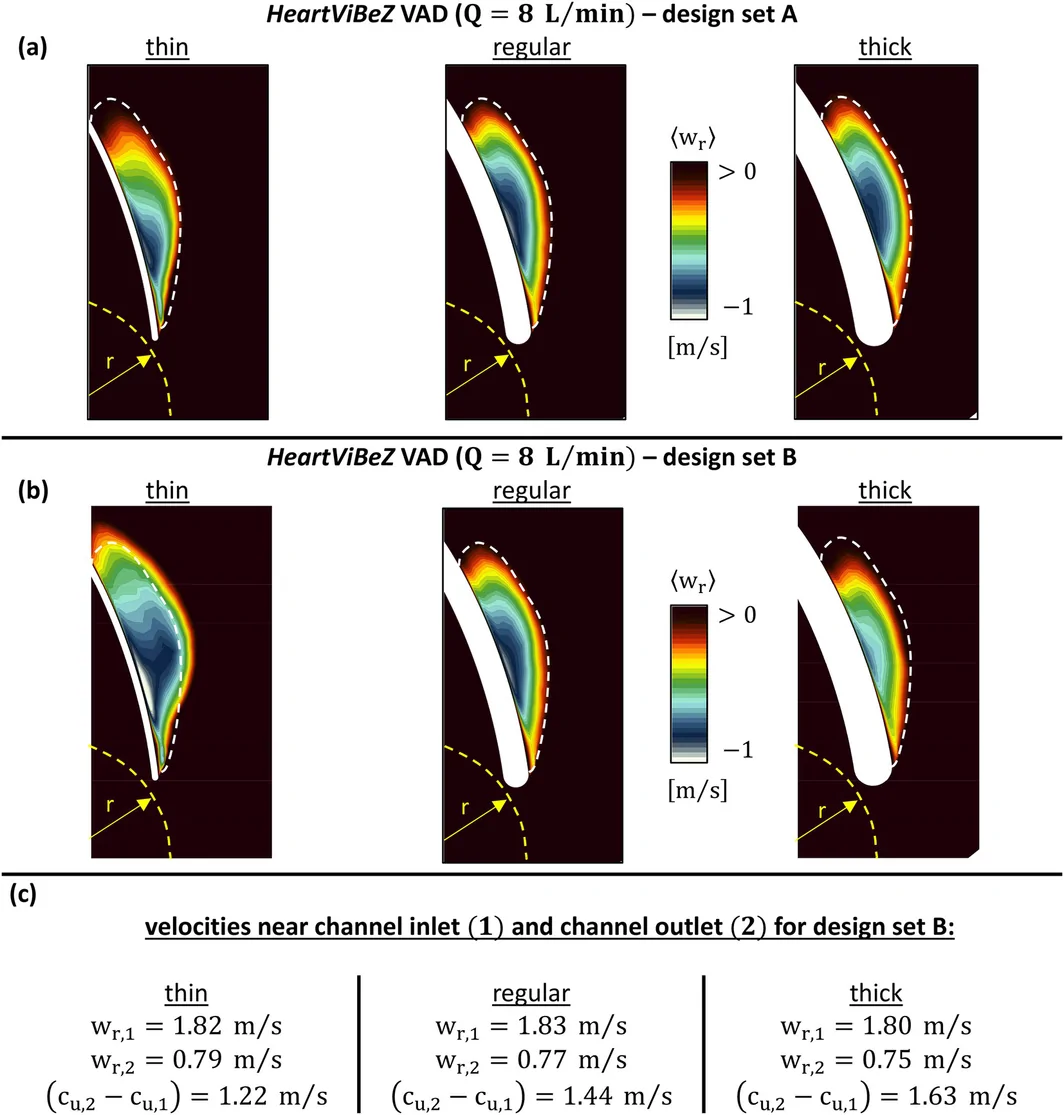
Local flow characteristics for the HeartViBeZ VAD at Q=8L/min. (a) Visualization of the separation behavior around the impeller blades using the radial velocity in the relative frame of reference for design set A. The white dotted line indicates zero radial velocity for the regular blade profile. (b) Corresponding visualization for design set B (adapted inlet and outlet blade channel geometries). (c) Averaged radial velocities near the channel inlet (1) and outlet (2), and circumferential velocity increment for design set B. w_r_—radial velocity in the relative frame of reference. c_u_—circumferential velocity in the absolute frame of reference.

As a second mechanism, the circumferential velocity increment $\left(c_{u,2}-c_{u,1}\right)$ increases with blade thickness, as shown in Figure 9c. This trend is consistent with enhanced flow guidance and pressure increase [10] with thicker blades, and provides a plausible explanation for the improved performance observed at overload conditions (Figure 8). However, both mechanisms appear secondary to the dominant blockage effect.

It should be noted that the corresponding trend observed for the *HeartViBeZ* VAD is less pronounced for the CPAD, for which no consistent performance improvement with increasing blade thickness is evident for design set B. This behavior likely reflects the complex interplay of multiple flow mechanisms affecting the remaining performance deviations in the CPAD, such as differences in flow deflection or friction loss distributions. It underscores that the relative importance of thickness‐related effects is pump‐specific when blockage effects are mitigated.

For both RBPs, variations in blade thickness were found to influence hemolysis metrics under off‐design conditions. However, the impact on damage index remains limited, with maximum relative deviations below 7%. This mirrors the comparatively small differences in flow losses observed between the investigated configurations. Since losses are directly related to the stresses in the flow [12], the relative deviations in DI are of similar magnitude to those observed for the losses (Figure 7).

As shown in Figures 6, 7, 8, no consistent trend indicating a universally favorable blade thickness is observed. While the thinner blades perform better under overload conditions for similar blade channel areas (design set A), they exhibit comparable or inferior performance at other conditions. This trade‐off provides a mechanistic explanation for the controversial findings reported in the literature [15, 16, 17, 18] regarding the effect of blade thickness in RBPs. In many of these studies, blade thickness effects were evaluated for a single pump and only over a limited number of operating points, which can lead to divergent conclusions. Moreover, the relative deviations in hemolysis are within a maximum range of approximately 10% for the investigated configurations. These magnitudes are consistent with the deviations observed in the present study, indicating that thickness‐induced effects on hemolysis remain generally limited in RBPs.

## 5 Limitations

The simulations were performed using the URANS method, which is subject to known limitations due to its modeling assumptions as well as spatial and temporal resolution. However, previous studies have shown that URANS‐based simulations are sufficient to capture the dominant hydraulic and hemolysis‐relevant mechanisms in rotary blood pumps [12]. Consequently, while the absolute values of quantities such as stresses, velocities, head, or efficiency may be affected by these limitations, the present study focuses on a comparative analysis of different blade‐shape configurations under identical numerical settings. Therefore, the numerical uncertainties are expected to influence all cases in a similar manner, and the observed trends and relative differences are therefore considered robust.

Hemolysis was quantified using a single power‐law formulation. Although this model is widely used, it does not fully capture the complex mechanisms of blood cell damage. Consequently, the presented hemolysis metric should be interpreted primarily in a comparative sense rather than as absolute predictors of hemolysis. This limitation is mitigated by the consistent use of the same hemolysis model.

The investigated blade thicknesses and leading‐edge geometries represent a clinically and technologically relevant design space for RBPs. However, the findings cannot be directly extrapolated to axial‐flow pumps. Accordingly, the conclusions of this study should be interpreted within the context of centrifugal RBP designs operating in clinically relevant flow regimes.

## 6 Conclusion

Taken together, the results demonstrate that impeller blade shape is not a primary driver of hemocompatibility in rotodynamic blood pumps (RBPs) within the investigated, clinically relevant operating ranges. Variations in leading‐edge geometry primarily affect local flow features, such as separation behavior and localized stress distributions, without translating into relevant changes in hydraulic performance or hemolysis‐related metrics.

Blade thickness, by contrast, represents a relevant design lever for off‐design hydraulic performance, particularly under overload conditions, where blockage effects within the blade channels dominate efficiency losses, while remaining of not primary importance for hemocompatibility.

Within the investigated parameter space, all examined leading‐edge geometries in combination with blade thicknesses between 0.25 and 1.50 mm appear to represent robust and design‐tolerant baseline design choices for centrifugal RBPs.

## Author Contributions

Conceptualization and study design were carried out by Marcus Granegger and Benjamin Torner. Methodology, data collection, and analysis were performed by Benjamin Torner, Simon Klocker, and Marcus Granegger. The CFD setup was developed by Benjamin Torner, Simon Klocker, Aldo J. Suria, and Niccolò Crescenti. Experimental validation was performed by Harald Reithofer and Aldo J. Suria. The first draft of the manuscript was written by Benjamin Torner, and all authors provided comments on earlier versions of the manuscript. Funding was obtained by Marcus Granegger, Daniel Zimpfer, and Michael Hübler. All authors read and approved the final manuscript.

## Funding

This work was supported by GIGAX Foundation, EVER Foundation, and Austrian Science Fund (10.55776/PIN5467323).

## Conflicts of Interest

Marcus Granegger received personal fees and grants from Berlin Heart GmbH. Daniel Zimpfer is proctor, advisor, and speaker (Medtronic Inc., Abbott Inc., Berlin Heart GmbH, Edwards, Abiomed), and received research and travel grants (Medtronic Inc. and Abbott Inc.).

## Supporting information


**Supporting Information: S1.** Experimental Validation Setup and Results for the Hydraulic Performance.

**Supporting Information: S2.** Grid Uncertainty Estimation for the 3 RBP Cases.

## Data Availability

The data that support the findings of this study are available from the corresponding author upon reasonable request.
