# Supplementary material for: Shaping the Flow: Impact of Blade Shape on Performance and Hemocompatibility in Rotodynamic Blood Pumps
Source: Artif Organs. 2026 Jun 7;50(10):1360–71. doi: 10.1111/aor.70178 (PMC13622911; doi:10.1111/aor.70178)
Supplement: Supplementary file 1 [file AOR-50-1360-s001.pdf]

Supplementary Material 1 – Experimental Validation Setup and Results for the Hydraulic Performance

In the following, the experimental test setups for the hydraulic performance measurements are displayed for the three rotodynamic blood pumps (RBPs). Additionally, the comparison between the experimentally measured and numerically assessed pressure head H – flow rate Q curves are shown.

Cavopulmonary Assist Device

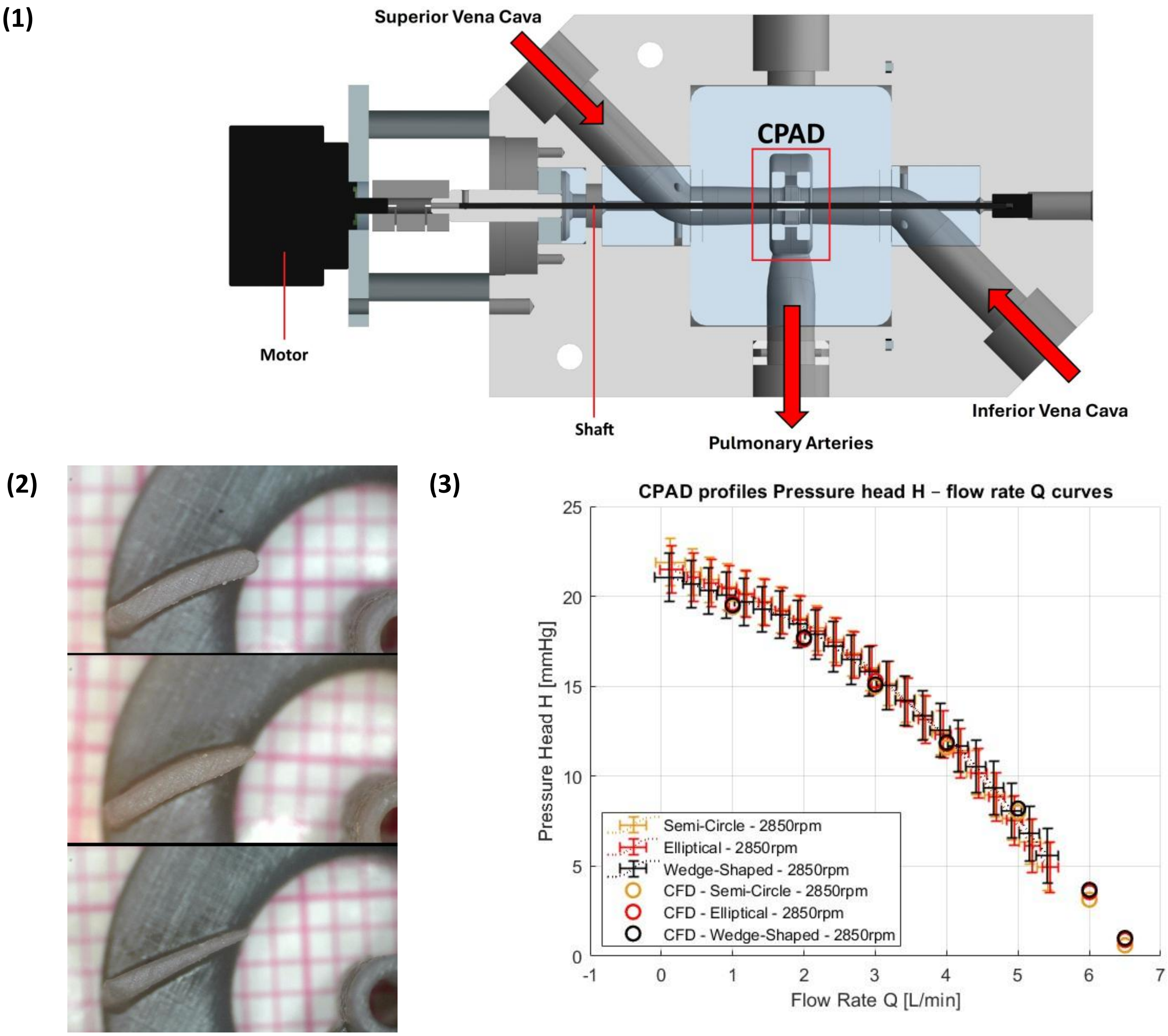

Figure A1: (1) Modular test rig for our externally driven cavopulmonary assist device (CPAD). (2) 3D-printed impellers of a CPAD with semi-circle, elliptical and wedge-shaped blade profiles. (3) Pressure head H – flow rate Q curves of semi-circle, elliptical and wedge-shaped blade profiles for the CPAD. Error bars show the ± uncertainty of the measurements, calculated in accordance with Ref. [31] from the main manuscript. N=3 for each impeller.

<u>Remark for the validation experiment for the CPAD</u>: Static pressures were measured in the testbench and were transformed to total pressures by adding the dynamic pressures in post processing. Since the experimental measurements were terminated at a static pressure of $0\ \mathrm{mmHg}$, the corrected total pressure curve does not extend to the zero-head condition, as no data was recorded beyond this point.

CentriMag Rotodynamic Blood Pump

(1)

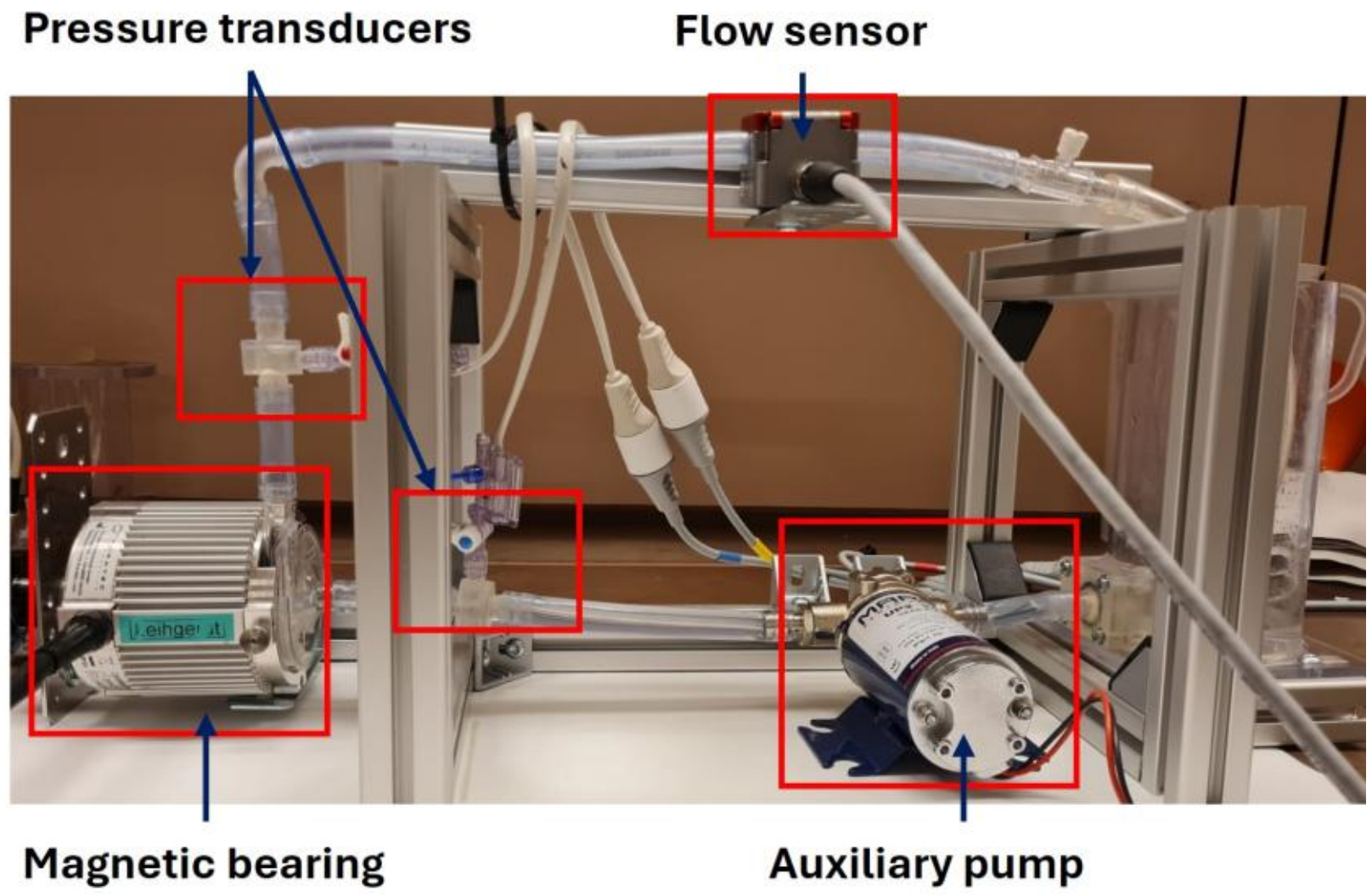

(2)

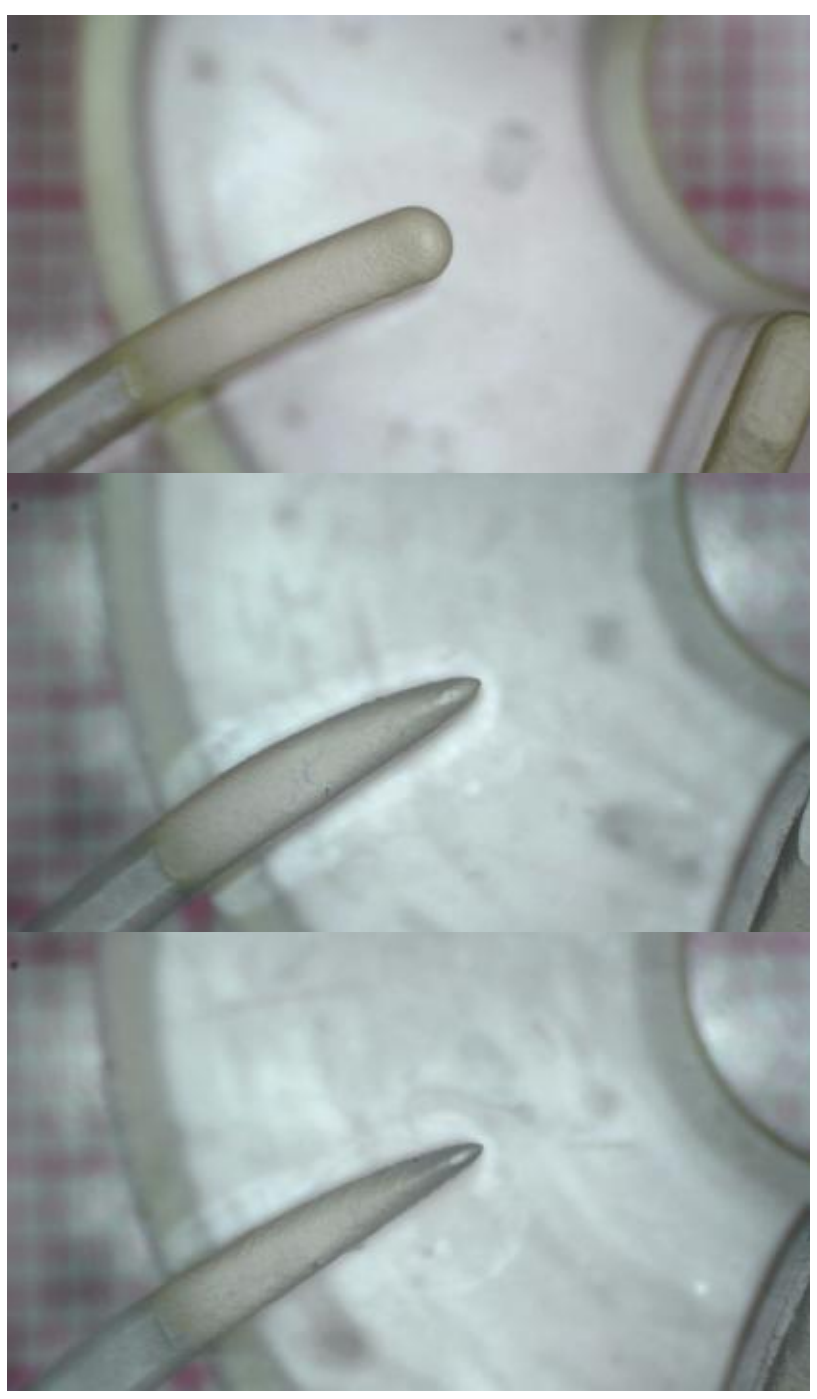

(3)

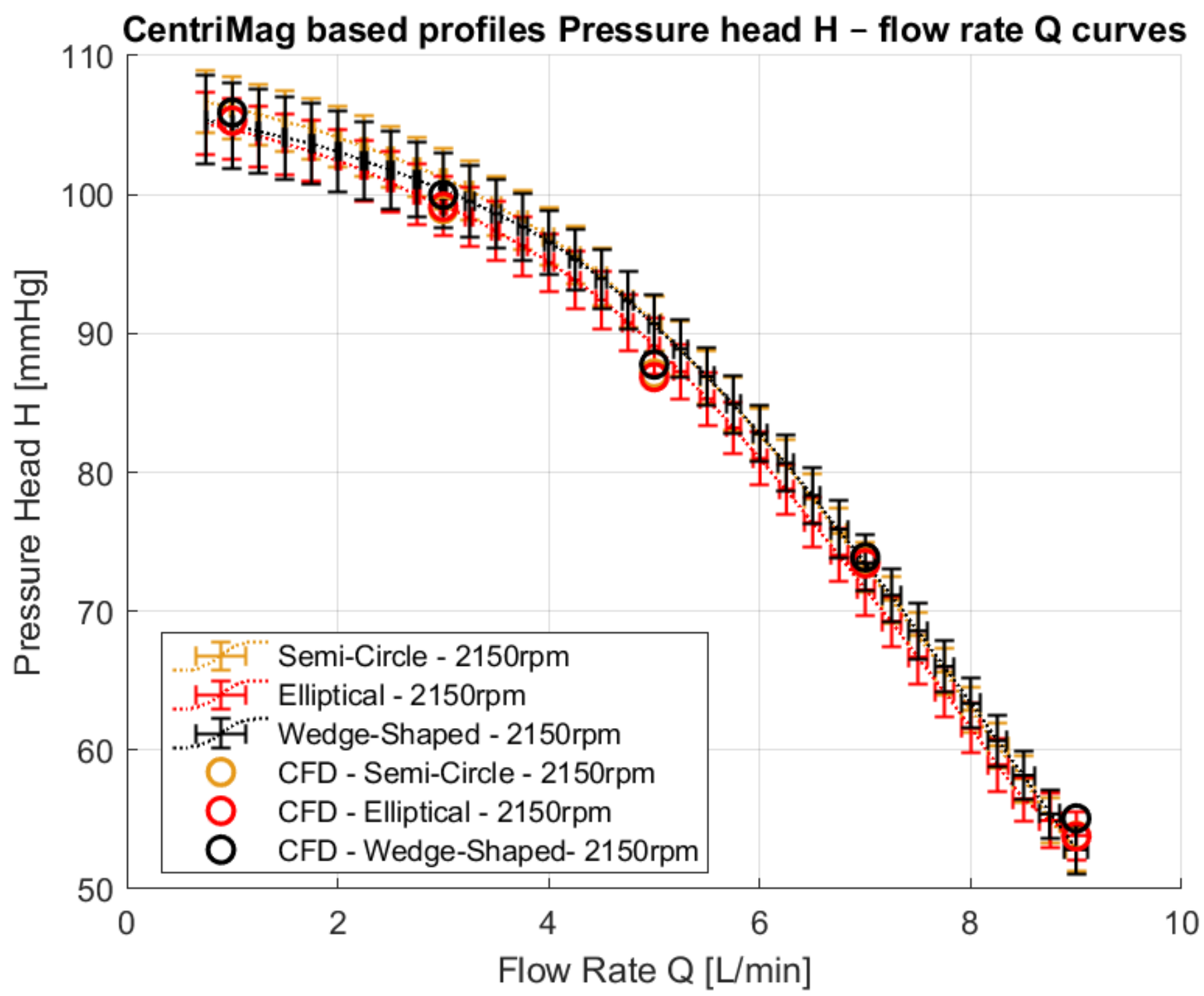

Figure A2: (1) Test rig for the magnetically levitated CentriMag rotodynamic blood pump (RBP). (2) 3D-printed impellers of the CentriMag RBP with semi-circle, elliptical and wedge-shaped blade profiles. (3) Pressure head H – flow rate Q curves of semi-circle, elliptical and wedge-shaped blade profiles for the CentriMag RBP. Error bars show the ± uncertainty of the measurements, calculated in accordance with Ref. [31] from the main manuscript. N=3 for each impeller.

Remark for the validation experiment for the CentriMag RBP: To enable a direct comparison between the experimentally obtained H–Q curves and the simulation results, the flow losses occurring in the test rig between the pressure transducers and the RBP were accounted for and subtracted from the simulated data. These losses comprised minor (local) losses due to area changes between the pressure transducers and the connecting tubes, which were computed by empirical relations. In addition, major losses caused by tube friction were calculated using the Darcy-Weisbach equation.

***HeartViBeZ* Ventricular Assist Device**

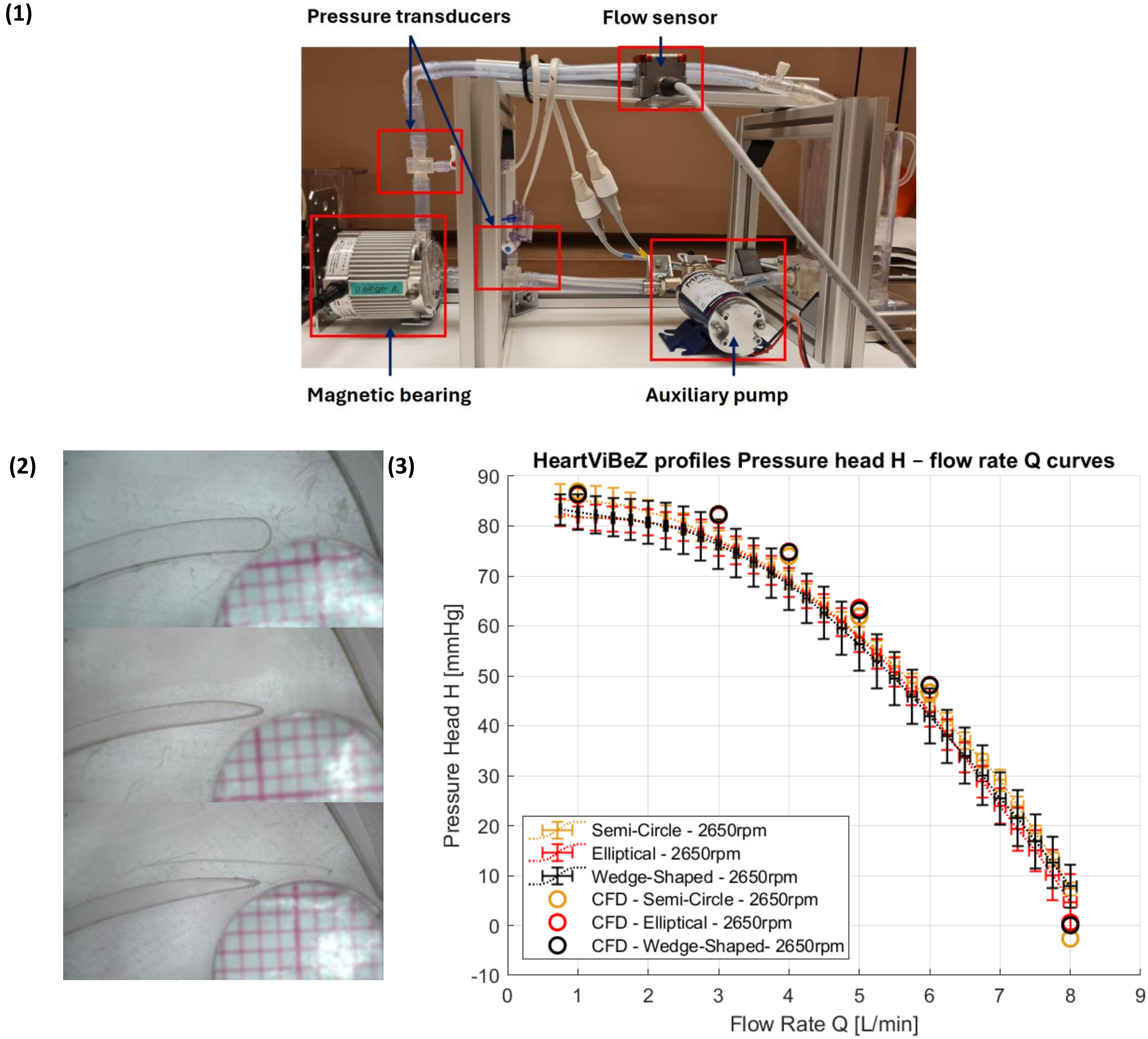

Figure A3: (1) Test rig for the magnetically levitated *HeartViBeZ* VAD. (2) 3D-printed impellers of the *HeartViBeZ* VAD with semi-circle, elliptical and wedge-shaped blade profiles. (3) Pressure head H – flow rate Q curves of the blade profiles. Error bars show the ± uncertainty of the measurements, calculated in accordance with Ref. [31] from the main manuscript. N=3 for each impeller.

Remark for the validation experiment for the *HeartViBeZ* VAD: To enable a direct comparison between the experimentally obtained H–Q curves and the simulation results, the flow losses occurring in the test rig between the pressure transducers and the RBP were accounted for and subtracted from the simulated data. These losses comprised minor (local) losses due to area changes between the pressure transducers and the connecting tubes, which were computed by empirical relations. In addition, major losses caused by tube friction were calculated using the Darcy-Weisbach equation.
