# Supplementary material for: Shaping the Flow: Impact of Blade Shape on Performance and Hemocompatibility in Rotodynamic Blood Pumps
Source: Artif Organs. 2026 Jun 7;50(10):1360–71. doi: 10.1111/aor.70178 (PMC13622911; doi:10.1111/aor.70178)
Supplement: Supplementary file 2 [file AOR-50-1360-s002.pdf]

Supplementary Material 2 – Grid Uncertainty Estimation for the 3 RBP Cases

Cavopulmonary Assist Device (CPAD)

In Figure B1, grid uncertainties are shown based on the estimation procedure reported in *"L. Eça, S. L. Toxopeus, and M. Kerkvliet, Procedures for the Estimation of Numerical Uncertainties in the Simulation of Steady and Unsteady Flows."* The variable h denotes a representative, global grid length, which is computed by $\mathrm{h} = \sqrt[3]{\mathrm{V}}/\mathrm{N_{grid}}$, with V as the mesh volume and $\mathrm{N_{grid}}$ as the grid cell number in this volume.

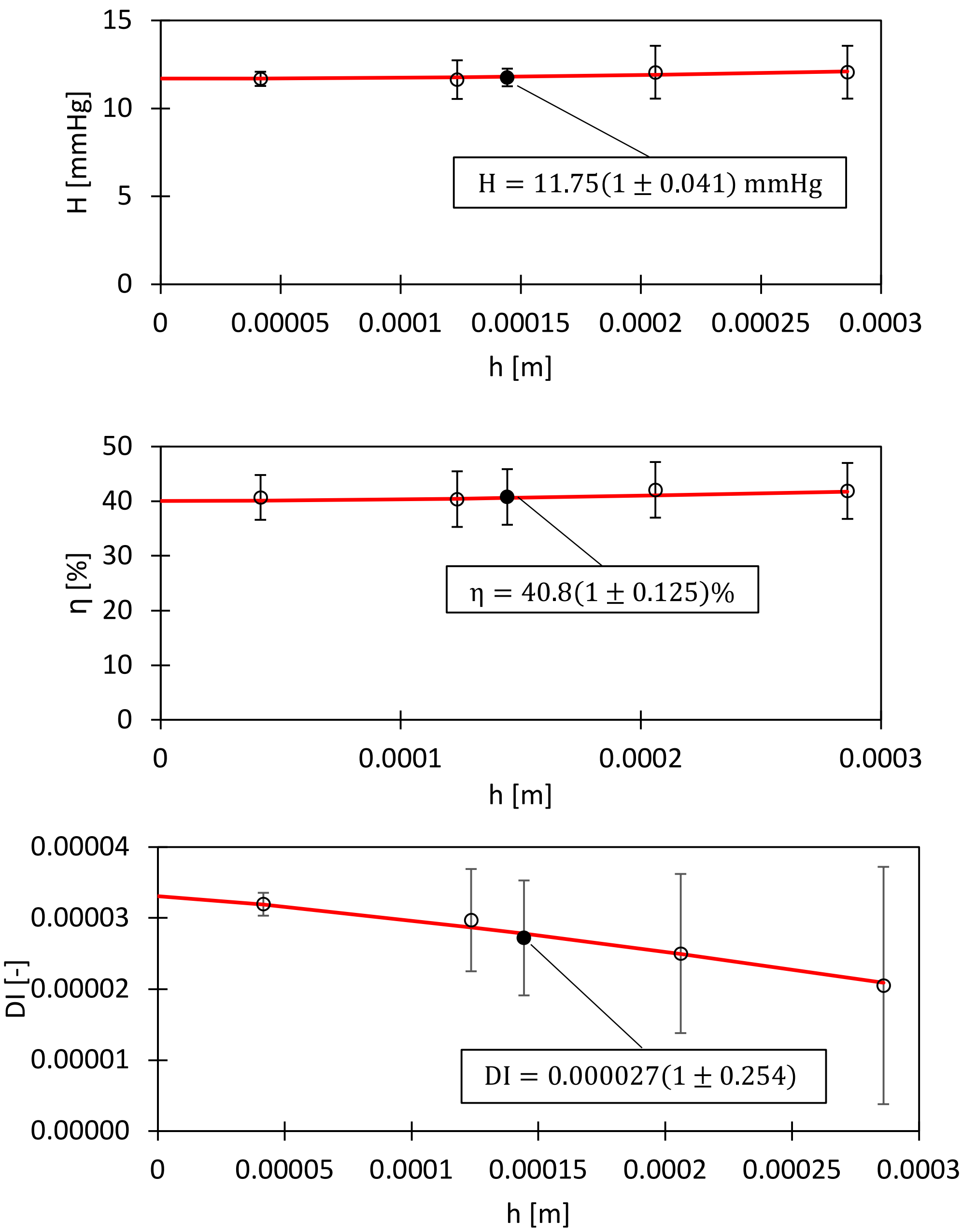

Figure B1: Grid uncertainty estimation for the simulation of the cavopulmonary assist device at the best efficiency operating condition at $n = 2850\ rpm$. (Top) Pressure head with uncertainties at different grids. (Middle) Efficiency with uncertainties at different grids. (Bottom) Damage index (DI) with uncertainties at different grids. The black dots indicate the results at the investigated grid used in this study. The red line shows the estimated fit of the data to the expectation value at $h = 0\ m$.

CentriMag Rotodynamic Blood Pump

In Figure B2, grid uncertainties are shown based on the estimation procedure reported in *"L. Eça, S. L. Toxopeus, and M. Kerkvliet, Procedures for the Estimation of Numerical Uncertainties in the Simulation of Steady and Unsteady Flows."* The variable h denotes a representative, global grid length, which is computed by $\mathrm{h} = \sqrt[3]{\mathrm{V}}/\mathrm{N}_{\mathrm{grid}}$, with V as the mesh volume and $\mathrm{N}_{\mathrm{grid}}$ as the grid cell number in this volume.

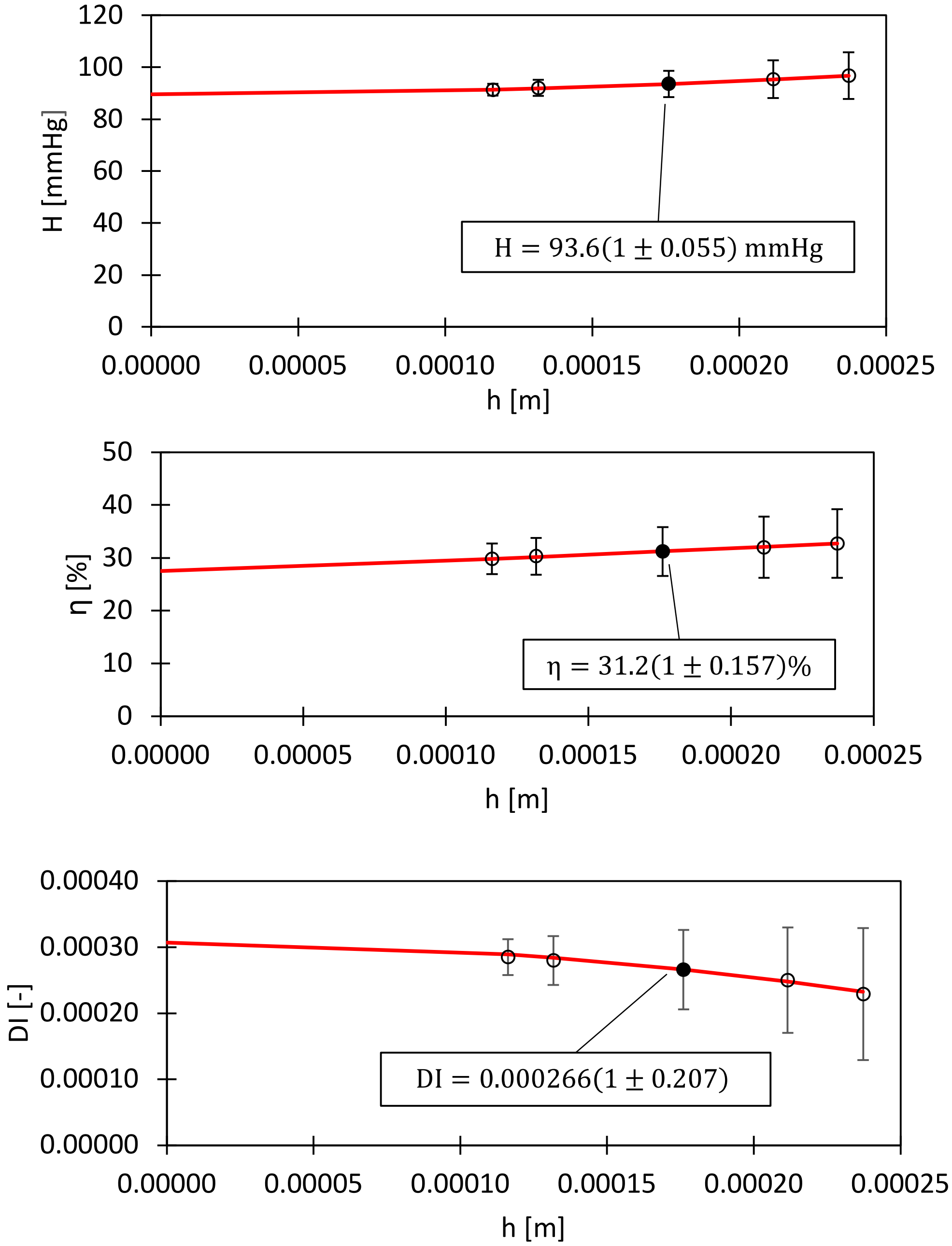

Figure B2: Grid uncertainty estimation for the simulation of the CentriMag rotodynamic blood pump at the best efficiency operating condition at $n = 2150\ rpm$. (Top) Pressure head with uncertainties at different grids. (Middle) Efficiency with uncertainties at different grids. (Bottom) Damage index (DI) with uncertainties at different grids. The black dots indicate the results at the investigated grid used in this study. The red line shows the estimated fit of the data to the expectation value at $h = 0\ m$.

HeartViBeZ Ventricular Assist Device

In Figure B3, grid uncertainties are shown based on the estimation procedure reported in *"L. Eça, S. L. Toxopeus, and M. Kerkvliet, Procedures for the Estimation of Numerical Uncertainties in the Simulation of Steady and Unsteady Flows."* The variable h denotes a representative, global grid length, which is computed by $\mathrm{h} = \sqrt[3]{\mathrm{V}}/\mathrm{N_{grid}}$, with V as the mesh volume and $\mathrm{N_{grid}}$ as the grid cell number in this volume.

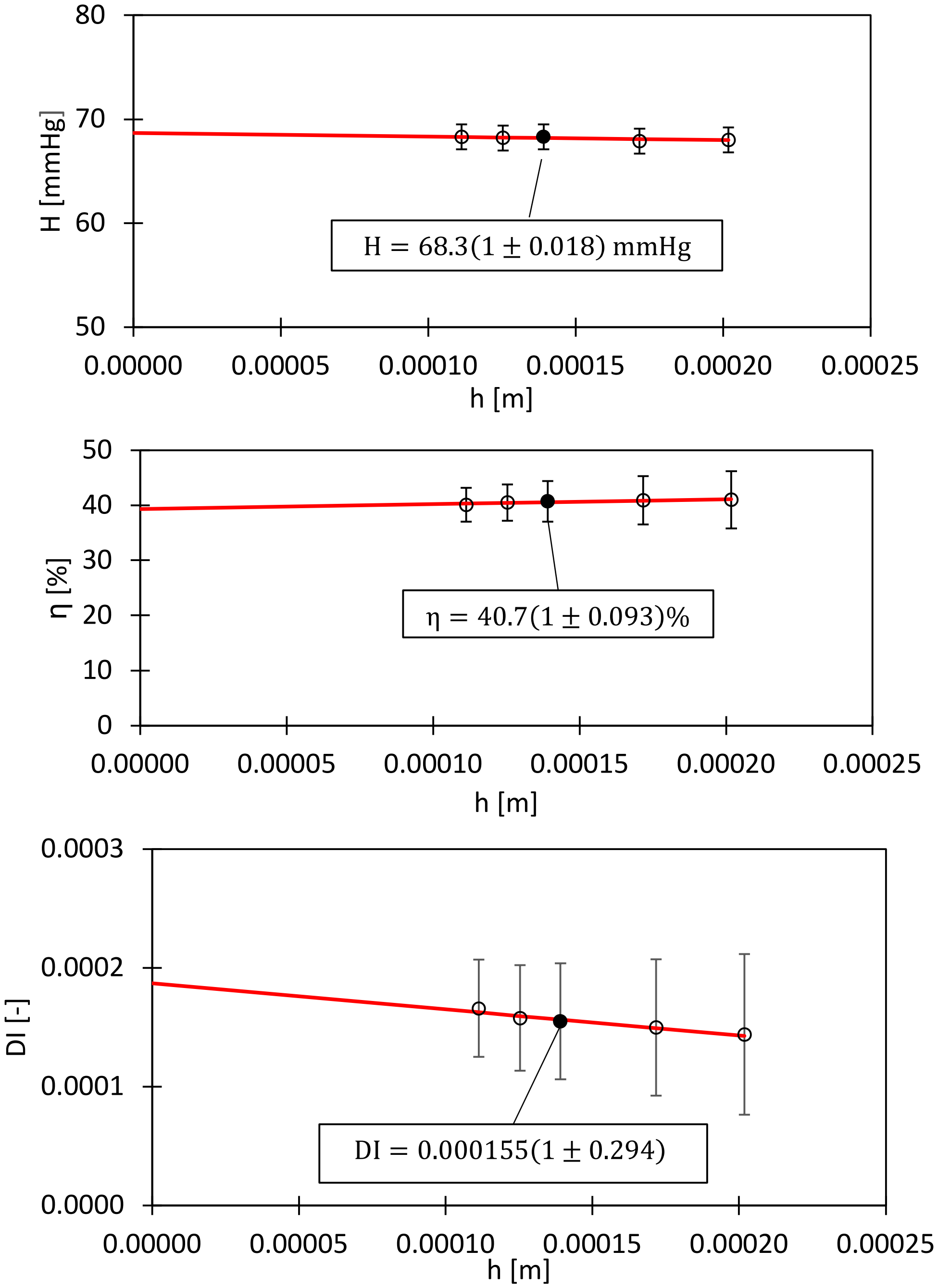

Figure B3: Grid uncertainty estimation for the simulation of the *HeartViBeZ* VAD at the best efficiency operating condition at $n = 2650\ rpm$. (Top) Pressure head with uncertainties at different grids. (Middle) Efficiency with uncertainties at different grids. (Bottom) Damage index (DI) with uncertainties at different grids. The black dots indicate the results at the investigated grid used in this study. The red line shows the estimated fit of the data to the expectation value at $h = 0\ m$.
